# The Nuclear Transcription Factor PKNOX2 Is a Candidate Gene for Substance Dependence in European-Origin Women

**DOI:** 10.1371/journal.pone.0016002

**Published:** 2011-01-27

**Authors:** Xiang Chen, Kelly Cho, Burton H. Singer, Heping Zhang

**Affiliations:** 1 Department of Epidemiology and Public Health, Yale University School of Medicine, New Haven, Connecticut, United States of America; 2 Emerging Pathogens Institute, University of Florida, Gainesville, Florida, United States of America; University of South Florida College of Medicine, United States of America

## Abstract

Substance dependence or addiction is a complex environmental and genetic disorder that results in serious health and socio-economic consequences. Multiple substance dependence categories together, rather than any one individual addiction outcome, may explain the genetic variability of such disorder. In our study, we defined a composite substance dependence phenotype derived from six individual diagnoses: addiction to nicotine, alcohol, marijuana, cocaine, opiates or other drugs as a whole. Using data from several genomewide case-control studies, we identified a strong (Odds ratio  = 1.77) and significant (p-value = 7E-8) association signal with a novel gene, PBX/knotted 1 homeobox 2 (PKNOX2), on chromosome 11 with the composite phenotype in European-origin women. The association signal is not as significant when individual outcomes for addiction are considered, or in males or African-origin population. Our findings underscore the importance of considering multiple addiction types and the importance of considering population and gender stratification when analyzing data with heterogeneous population.

## Introduction

Substance dependence or addiction is one of the most sought-after phenomena in many populations because of its serious health and socio-economic consequences. In 2008, the Centers for Disease Control estimated that 443,000 deaths were caused by cigarette smoking and exposure to secondhand smoke [1]. In addition, alcohol misuse has been linked to attempted and successful suicide, particularly among adolescents [2]. There is strong evidence that vulnerability to substance dependence to drugs, alcohol, or smoking is a complex trait with both genetic and environmental components [3], [4], [5]. Therefore, a better understanding of the genetics behind vulnerability to addictions could tremendously improve overall health and quality of life in general. A useful start in this direction is given by Kreek et al. [6] In the literature, candidate genes for addiction to individual substances (alcohol, nicotine and other substances) have been identified. For example, well studied genes for alcohol dependence, such as GABRA2, CHRM2 and ADH4, have been replicated in many samples [7], [8], [9], [10], while several newer candidate genes (GABRG3, TAS2R16, SNCA, OPRK1 and PDYN) remain to be confirmed [11]. Multiple variants at the aldehyde dehydrogenase (ALDH) and alcohol dehydrogenase (ADH) loci have also been well documented as genes of major genetic effect especially in East-Asian populations [12], [13], [14], [15]. A gene cluster of nicotinic acetylcholine receptors (CHRNA5, CHRNA3, and CHRNB4) and Neurexin1, also show allelic differences in heavy vs. light smokers in multiple studies [16], [17], [18], [19]. Li [20] reported thirteen regions on chromosomes 3–7, 9–11, 17, 20, and 22, to be significantly associated with nicotine dependence in at least two independent samples, although a significant number of reported genomic regions did not reach the level of “suggestive” or “significant” linkage and failed to be replicated in other independent studies.

In the past, much effort has been devoted to the emphasis on individually different substance dependence outcomes. However, substance dependence as a whole, combining addiction to nicotine, alcohol, marijuana, cocaine, opiates and other drugs, has not been thoroughly investigated in association studies. A composite substance dependence phenotype may be the key to finding a common genetic predisposition of substance dependence as a whole. This common genetic predisposition may not be apparent when individual addiction conditions are considered. In the literature, Li and Burmeister [21] provide a good review of comorbidity in the genetics of addiction. The availability of the Gene Environment Association Studies Genes and Environment Initiative Study of Addiction: Genetics and Environment (SAGE) data provides an unprecedented opportunity to study the genetics of a composite trait: namely, addiction to at least two of the six substances under study (nicotine, alcohol, marijuana, cocaine, opiates and other drugs).

In this report, we present a genomewide significant association (α = 0.05) of PKNOX2 gene on chromosome 11 with composite substance dependence in European-origin women. We have identified a cluster of markers in the region of PKNOX2 gene that are strongly associated with a composite addiction phenotype rather than with any single addiction type. Furthermore, we investigate potential sex-specificity and racial differences in the association. The nuclear transcription factor PKNOX2 has been previously identified as one of the *cis*-regulated genes for alcohol addiction in mice [22]. However, to our knowledge, PKNOX2 has not been reported to be associated with any substance addiction outcomes in human populations. Thus we present PKNOX2 as a novel candidate gene for substance dependence in humans.

## Methods

### Study of Addiction: Genetics and Environment (SAGE) Data

We obtained the genomewide single nucleotide polymorphisms (SNP) data from the database of Genotype and Phenotype (dbGaP). The data were from the Study of Addiction: Genetics and Environment (SAGE) (Bierut et al. 2010). We included 4,121 subjects for whom the addiction to the six categories of substances and genomewide SNP data (ILLUMINA Human 1M platform) were available. SAGE is a case-control study of mostly unrelated individuals aimed at identifying genetic associations for addiction. Cases and controls were selected from three large, complementary cohorts: Collaborative Study on the Genetics of Alcoholism (COGA, initiated in 1989), Family Study of Cocaine Dependence (FSCD, 2000–2006), and Collaborative Genetic Study of Nicotine Dependence (COGEND, initiated in 2000). These three studies have been previously described [8], [23], [24], [25]. Lifetime dependence on nicotine, alcohol, marijuana, cocaine, opiates or other dependence on other drugs was diagnosed in accordance with the Diagnostic and Statistical Manual of Mental Disorders, Fourth Edition (DSM-IV). As stated above, we studied a composite addiction phenotype according to whether a subject was addicted to substances in at least two of the aforementioned categories.

### Subject Characteristics and Study Design

To reduce the level of noise in genotypes and increase the efficiency of analysis, we filtered SNPs by setting thresholds for minor allele frequency (MAF) and call rate (i.e., MAF >5% and call rate >90%). In addition, we excluded 60 duplicate genotype samples and removed 9 subjects with ethnic backgrounds other than African-origin (Black) or European-origin (White). Table 1 lists the descriptive statistics of the sample included in our study. Almost all subjects were either Black (30.3%) or White (69.4%).

**Table 1:** Descriptive statistics of the sample stratified by sex and race.

	Black Men	White Men	Black Women	White Women	Overall
n	535	1131	568	1393	3627
Age (SD) yr.	40.9 (8.2)	38.7 (10.3)	39.7 (6.7)	38.2 (9.1)	39.0 (9.1)
Height (SD) m	1.78 (0.08)	1.79 (0.07)	1.64 (0.08)	1.65 (0.07)	1.71 (0.10)
Weight (SD) kg	89.7 (18.3)	88.7 (16.8)	85.0 (21.9)	72.4 (18.8)	81.1 (20.1)
Alcohol (%)	62.1	62.3	39.4	31.1	46.7
Cocaine (%)	46.4	27.3	36.3	12.5	25.8
Marijuana (%)	25.4	25.2	13.7	8.7	17.1
Nicotine (%)	47.5	46.7	47.7	41.1	44.8
Opiates (%)	8.2	9.9	6.2	4.8	7.1
Other drugs (%)	11.4	18.0	6.5	9.4	11.9
No drug (%)	27.1	31.0	38.9	50.1	39.0

**Figure 1:**
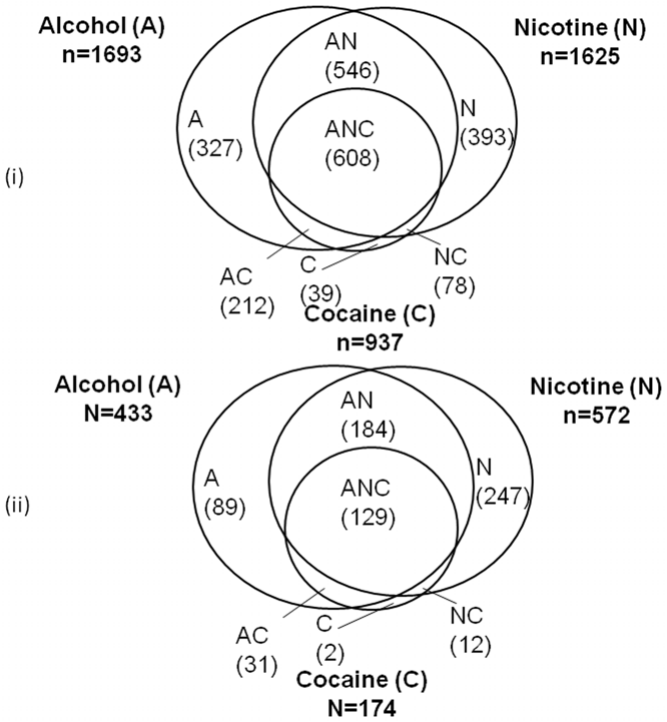
Number of substance dependent subjects according to DSM-IV for the top three addiction categories: alcohol (A), nicotine (N) and cocaine (C). (i) is based on the overall sample and (ii) is based on the White women subset.

In our final analysis, a total of 3,627 unrelated subjects with 830,696 autosomal SNPs were included. The final subset considered in the analysis consisted of 45.9% men and 54.1% women with mean ages of 39.4 and 38.6 years old, respectively. Because substance dependence is a complex disease with both genetic and environmental components, we analyzed the male and female subsets separately. In addition, we performed separate analysis for Blacks and Whites in light of the possibility that underlying genetic variants may be different in different ethnic groups. Thus our study included four sub-samples: 1,393 White women, 1,131 White men, 568 Black women and 535 Black men. Overall a total of 1,513 subjects were defined as having two or more substance addictions according to DSM-IV. Of these, there were 316, 585, 237 and 375 subjects in the Black male, White male, Black female and White female subsets, respectively. The proportions of subjects diagnosed with lifetime dependence on substances in each of the six categories – nicotine, alcohol, marijuana, cocaine, opiates or other drugs – are presented in Table 1. The top three most widely used substances among the six were alcohol, nicotine and cocaine, in that order (Figure 1).

### Statistical Analysis

We took precaution to investigate and control for potential confounding by stratification and admixture due to disequilibrium between pairs of unlinked loci [26] given the different ethnic populations in our data. To avoid potential population stratification, we first stratified our analysis by race and sex. Then we performed formal population stratification analysis for each subset using PLINK software (version 1.04) [27]. The results confirmed that each subset comes from a homogenous population; thus no further adjustment was needed to control for potential confounding by stratification and admixture.

We used allelic Chi-square tests with 1 degree of freedom in our analysis, stratified by race and sex. Haploview (version 4.0) [28] was used to analyze the linkage disequilbrium in the PKNOX2 gene region and the association between the haplotypes and the composite phenotype. We performed additional analyses by examining and comparing the results of including and excluding 214 related subjects in the data. For mixtures of unrelated and related subjects, we used PedGenie [29] to perform association analyses. PedGenie first performs the allelic Chi-square tests treating all individuals independently, then takes the pedigree information into account to assess statistical significance through permutation analysis.

### Determination of Significance Threshold for GWAS

Using genotypes from the Wellcome Trust Case-Control Consortium, Dudbridge and Gusnanto [30] studied the genomewide significance threshold for the UK Caucasian population. They subsampled the genotypes at different densities and estimated the threshold for 5% family-wise error using permutations. They then extrapolated to infinity density and estimated that the genomewide significance threshold for this population is 7.2E-8. We used this genomewide significance threshold (7.2E-8) for the Caucasian population (white men and white women) in our analysis.

## Results

The top 8 significant SNPs are summarized in Table 2. They cluster in PKNOX2 on chromosome 11 (11q24). None of the 8 SNPs violates the Hardy Weinberg equilibrium assumption (minimum *p*-level  = 0.12). Among them, rs12284594 is the most significant SNP (p-value = 7.13E-08) observed in White women with an odds ratio (OR) of 1.77, suggesting that those who have the risk allele (G) for rs12284594 are at significantly increased risk of being diagnosed with at least two of the six categories of substance dependence. This p-value reaches the accepted genomewide significance level [30]. In addition, there are 7 other SNPs with p-values less than 3.8E-06 in White women with similar ORs (1.63 - 1.72). We further examined association of haplotypes with the composite phenotype in this region, but they did not enhance the strength of the associations; hence these results are not reported here. Similarly, when related individuals were included in the analysis, the strength of association was not enhanced, whether the analysis was performed using PedGenie [29], or whether the correlation among related individuals was ignored (data not shown). Although we also observed that these 8 SNPs confer increased risk in White men, Black men and Black women, they fail to reach genomewide significance. Hence, detailed results are not presented here for these groups.

**Table 2:** Summary of the 8 most significant SNPs in PKNOX2 gene showing genomewide significant association with substance dependence in White women.

SNP	P-value	OR
rs1426153 (G)	1.84E-06	1.66
rs11220015(A)	1.97E-06	1.65
rs11602925(G)	1.24E-06	1.67
rs750338(C)	4.22E-07	1.63
rs12273605(T)	3.83E-06	1.71
rs10893365(C)	2.27E-07	1.72
rs10893366(T)	6.87E-07	1.70
**rs12284594(G)**	**7.13E-08**	**1.77**

The high-risk allele is in the parenthesis.

We performed additional analyses to examine each substance dependence outcome separately for the top 8 SNPs presented above. Table 3 shows the corresponding p-values for the 8 SNPs for each substance dependence outcome. Alcohol dependence shows the strongest association (p-value = 1.97E-6 with rs12284594); however none of these p-values attains the genomewide significance level of 0.05.

**Table 3:** Associations of the 8 most significant SNPs in PKNOX2 with six individual substance dependence outcomes (p-values).

SNP	Nicotine	Alcohol	Marijuana	Cocaine	Opiates	Others
rs1426153 (G)	0.0159	5.75E-5	7E-4	3E-4	0.0113	1E-4
rs11220015(A)	0.0163	6.86E-5	0.0010	3E-4	0.0037	4.18E-5
rs11602925(G)	0.0136	4.24E-5	7E-4	3E-4	0.0059	5.29E-5
rs750338(C)	0.0491	4.26E-5	0.0013	2E-4	0.0112	2.22E-5
rs12273605(T)	0.0921	3E-4	3.53E-5	1E-4	0.0680	3.11E-5
rs10893365(C)	0.0411	1.72E-5	8.58E-6	2.91E-5	0.0699	2.58E-5
rs10893366(T)	0.0621	1.37E-5	8.80E-6	8.63E-5	0.0905	5.35E-5
**rs12284594(G)**	0.0239	1.97E-6	8.54E-6	4.39E-5	0.0533	2.45E-5

## Discussion

We have found a novel, genomewide significant association of a composite substance dependence phenotype with a SNP in the PKNOX2 gene in White women. PKNOX2, PBX/knotted 1 homeobox 2, belongs to the three-amino-acid loop extension (TALE) homeobox family. Homeodomain proteins are highly conserved transcription regulators. Imoto et al. [31] identified PKNOX2 as a novel TALE homeodomain-encoding gene, located at 11q24 in humans and it functions as a nuclear transcription factor indicated by its structure and sub-cellular localization. Later, PKNOX2 was identified as one of the *cis*-regulated genes for alcohol addiction in mice [22]. However, PKNOX2 has not been reported to be associated with any substance dependence phenotype in humans to date.

The composite dichotomous substance dependence variable reflects cases with two or more addictions where the top three categories are alcohol, nicotine and cocaine (Figure 1). Among the cases, 47% have been diagnosed with alcohol dependence in combination with other substance dependence outcomes. Our results, which show a strong association of this composite substance dependence variable with PKNOX2 gene in a human sample, support the experimental findings in mice by Mulligan et al [22]. Thus our findings make an important contribution in reporting PKNOX2 as a novel candidate gene for substance dependence in humans, particularly for White women in the SAGE sample.

Interestingly, among our most significant SNPs, we do not observe those genes previously reported for alcoholism or nicotine. Rather we find a new set of genes among the top SNPs. When each substance dependence outcome was individually analyzed for association with the 8 most significant SNPs, we found no association that reached the genomewide significance. This suggests that substance dependence or addiction as a whole has different risk genes compared to any single addiction outcome. It may also mean that there is more power in detecting common genes acting upon co-morbid addiction outcomes as a whole.

For many complex diseases, different ethnic groups have vastly different underlying genetics, and these differences may confound association results when they are pooled together as one in the analysis. Previously, racial differences in the prevalence of substance abuse have been reported [32], [33], [34]. More recently, Luo et al. [24] have reported that genetic differences between Black smokers and White smokers influence the nature of their nicotine dependence. Their analysis suggested that Black smokers become dependent at a lower threshold (number cigarettes per day) than Whites. On the other side, in the presence of subjects in different ethnic populations in the data, it is crucial to investigate and control for potential confounding by stratification and admixture due to disequilibrium between pairs of unlinked loci [26]. Thus we investigated these two major ethnic groups separately in our analysis. In addition, we stratified our analysis by gender; based on the premise that gender may be a confounding factor for the substance dependence outcome – men may be socially more prone to environmental influences promoting substance use, and thus more vulnerable to addiction, compared to women [35]. Our results from the two ethnic groups do not corroborate each other, which underscores the underlying genetic differences in White and Black samples. In fact, strong association signals are observed only in the White woman sample. With a heterogeneous population like SAGE, one must be cautious in analyzing and interpreting the results.

The identification of PKNOX2 as a candidate gene for substance use disorders underscores two important issues: (a) this has not been possible in the past due to limited sample size; and (b) we have considered a composite trait of six substance dependence outcomes as a whole. The association becomes less significant if individual substance addictions are considered. Thus, this result highlights the importance of studying highly comorbid disorders or those which might otherwise have a common pathway. However, our study is limited to the information in the available data, and we acknowledge the difficulty in operationalizing substance dependence; whether our operationalization of addiction to two or more substances, truly reflects the strength of the addiction phenotype is open to question. Indeed, it may simply reflect the extent of access to drugs. We also recognize that dependence on one substance shows different characteristics from dependence on another, and it is valuable and necessary to study them as individual entities. However, our call for more attention to comorbidity and the combinatorial study of these disorders should be viewed as a valuable complementary effort.
